# Study on the Synthesis, Surface Activity, and Self-Assembly Behavior of Anionic Non-Ionic Gemini Surfactants

**DOI:** 10.3390/molecules29081725

**Published:** 2024-04-11

**Authors:** Zhiqiang Man, Wenxiang Wu

**Affiliations:** 1 Key Laboratory of Enhanced Oil Recovery, Northeast Petroleum University, Ministry of Education, Daqing 163318, China; 2 No. 1 Oil Production Plant, PetroChina Daqing Oilfield Company, Daqing 163001, China

**Keywords:** negative non-ionic Gemini surfactant, self-assembly behavior, surface tension, thermodynamic parameters, oil recovery

## Abstract

The use of surfactants in oil recovery can effectively improve crude oil recovery rate. Due to the enhanced salt and temperature resistance of surfactant molecules by non-ionic chain segments, anionic groups have good emulsifying stability. Currently, there are many studies on anionic non-ionic surfactants for oil recovery in China, but there is relatively little systematic research on introducing EOs into hydrophobic alkyl chains, especially on their self-assembly behavior. This article proposes a simple and effective synthesis method, using 3-aminopropane sulfonic acid, fatty alcohol polyoxyethylene ether, and epichlorohydrin as raw materials, to insert EO into hydrophobic alkyl chains and synthesize a series of new anionic non-ionic Gemini surfactants (CnEO-5, *n* = 8, 12, 16). The surface activity, thermodynamic properties, and self-assembly behavior of these surfactants were systematically studied through surface tension, conductivity, steady-state fluorescence probes, transmission electron microscopy, and molecular dynamics simulations. The surface tension test results show that CnEO-5 has high surface activity and is higher than traditional single chain surfactants and structurally similar anionic non-ionic Gemini surfactants. Additionally, thermodynamic parameters (e.g., *ΔG°_mic_ ΔH°_mic_ ΔS°_mic_* et al. indicate that CnEO-5 molecules are exothermic and spontaneous during the micellization process. DLS, *p*-values, and TEM results indicate that anionic non-ionic Gemini surfactants with shorter hydrophobic chains (such as C8EO-5) tend to form larger vesicles in aqueous solutions, which are formed in a tail to tail and staggered manner; Negative non-ionic Gemini surfactants with longer hydrophobic chains (such as C12EO-5, C16EO-5) tend to form small micelles. The test results indicate that CnEO-5 anionic non-ionic Gemini surfactants have certain application prospects in improving crude oil recovery.

## 1. Introduction

Research has shown that surfactants used for oil recovery are very important for oil extraction, It can effectively improve crude oil recovery rate. Surfactant oil displacement technology has broad research prospects. In recent years, a series of new advances have been made in the research of surfactants for oil recovery. Gemini surfactants are generally composed of two monomer surfactant molecules connected by spacer units, and are therefore also known as dimeric surfactants [1].

Compared to single chain surfactants, these surfactants have higher surface activity, lower Kraft characteristics, stronger emulsifying and interfacial adsorption properties, and have broader application prospects in fields such as tertiary oil recovery, sterilization, and metal corrosion prevention [2,3,4]. This has also led to further research on the influence of surfactant molecules on their performance [5,6]. The hydrophobic groups of Gemini surfactants are generally long and straight carbon chains. Inserting different functional groups into the hydrophobic alkyl chains may improve their surface activity and application range [7,8,9]. Ma et al. introduced EOs functional groups into anionic head groups to synthesize anionic non-ionic Gemini surfactants with lower interfacial tension and wetting reversal properties [10]. Chen et al. found through molecular dynamics simulation that introducing EOs into anionic hydrophilic groups can further enhance the system’s tolerance to Ca^2+^ [11].

At present, there is relatively little systematic research on the introduction of EOs into hydrophobic alkyl chains, especially on their self-assembly behavior. This article synthesized a series of novel anionic non-ionic Gemini surfactants (CnEO-5, *n* = 8, 12, 16) by inserting EO into hydrophobic alkyl chains using 3-aminopropane sulfonic acid, fatty alcohol polyoxyethylene ether, and epichlorohydrin as raw materials. Then, the surface activity, thermodynamic properties, and self-assembly behavior of these surfactants were systematically studied through surface tension, conductivity, steady-state fluorescence probes, transmission electron microscopy, and molecular dynamics simulations. To our knowledge, this is the first systematic study on the introduction of EOs into hydrophobic alkyl chains, particularly on their self-assembly behavior.

## 2. Results and Discussion

### 2.1. Structural Characterization

**Figure 1:**
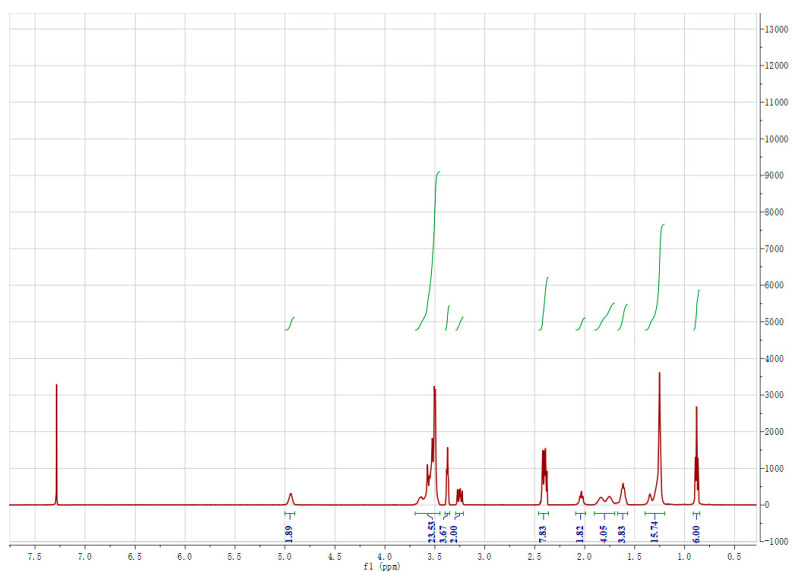
Integrated curve of C_8_EO-5 nuclear magnetic resonance hydrogen spectrum.

The C_n_EO-5 molecule was characterized by 1H NMR and elemental analysis. Figure 1 shows the integration curve of C_8_EO-5 nuclear magnetic resonance hydrogen spectrum:

From the integrated data in the figure, it can be seen that the number of hydrogen obtained from the integrated area of the nuclear magnetic resonance hydrogen spectrum is basically consistent with the number of hydrogen in C8EO-5.

**Figure 2:**
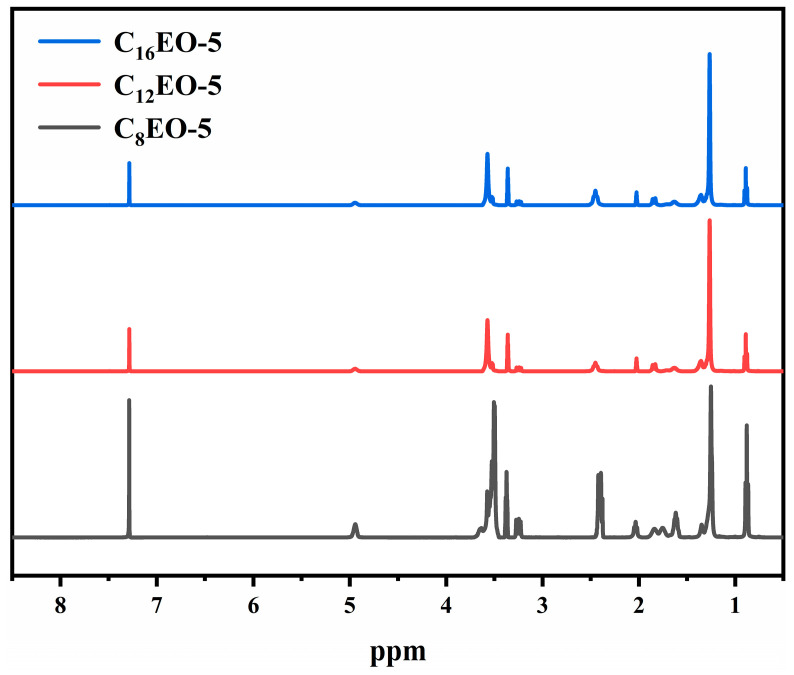
CnEO-5 Nuclear Magnetic Resonance Hydrogen Spectrogram.

Figure 2 shows the nuclear magnetic resonance hydrogen spectra of three synthesized surfactants:

The analysis of nuclear magnetic resonance hydrogen spectrum and elemental analysis results is as follows:

C8EO-5: 1H NMR (400 MHz, CDCl3, TMS) δ ppm: d: 0.84–0.94(t, 6H, CH2-CH3), 1.18–1.41 (m, 16H, CH2-CH3), 1.56–1.69 (m, 4H, CH3-(CH2)4-CH2), 1.69–1.90 (m, 4H, CH2-CH2-CH2-O), 1.9–2.10 (m, 2H, N-CH2-CH2), 2.35–2.44 (m, 8H, CH2-CH2-SO3, N-(CH2)3), 3.21–3.28 (m, 2H, CH-CH2-OH), 3.35–3.39 (m, 4H, CH2-CH2-CH2-O), 3.44–3.69 (m, 24H, O-CH2-CH2-O, CH-CH2-OH), 4.89–4.98 (s, 2H, CH-CH2-OH). Anal. Calcd. for C45H92NO17SNa %: C, 55.48; H, 9.52; N, 1.44; S, 3.29. Found %: C, 55.39; H, 9.63; N, 1.41; S, 3.27.
C12EO-5: 1H NMR (400 MHz, CDCl3, TMS) δ ppm: d: 0.85–0.91(t, 6H, CH2-CH3), 1.20–1.42 (m, 32H, CH2-CH3), 1.56–1.71 (m, 4H, CH3-(CH2)4-CH2), 1.78–1.89 (m, 4H, CH2-CH2-CH2-O), 1.99–2.06 (m, 2H, N-CH2-CH2), 2.36–2.52 (m, 8H, CH2-CH2-SO3, N-(CH2)3), 3.20–3.29 (m, 2H, CH-CH2-OH), 3.32–3.40 (m, 4H, CH2-CH2-CH2-O), 3.46–3.63 (m, 24H, O-CH2-CH2-O, CH-CH2-OH), 4.87–4.99(s, 2H, CH-CH2-OH). Anal. Calcd. for C45H92NO17SNa %: C, 55.48; H, 9.52; N, 1.44; S, 3.29. Found %: C, 55.39; H, 9.63; N, 1.41; S, 3.27.
C16EO-5: 1H NMR (400 MHz, CDCl3, TMS) δ ppm: d: 0.81–0.93 (t, 6H, CH2-CH3), 1.20–1.42 (m, 48H, CH2-CH3), 1.55–1.72 (m, 4H, CH3-(CH2)4-CH2), 1.69–1.90 (m, 4H, CH2-CH2-CH2-O), 1.97–2.10 (m, 2H, N-CH2-CH2), 2.34–2.51 (m, 8H, CH2-CH2-SO3, N-(CH2)3), 3.18–3.29 (m, 2H, CH-CH2-OH), 3.30–3.38 (m, 4H, CH2-CH2-CH2-O), 3.43–3.62 (m, 24H, O-CH2-CH2-O, CH-CH2-OH), 4.86–4.97 (s, 2H, CH-CH2-OH). Anal. Calcd. for C60H122NO17SNa %: C, 60.83; H, 10.38; N, 1.18; S, 2.71. Found %: C, 60.77; H, 10.42; N, 1.19; S, 2.69.
Due to the difference in the length of hydrophobic alkyl chains among the three surfactants, the chemical shifts of some characteristic functional groups (such as EO, sulfonic acid ions, etc.) in 1H NMR do not show significant changes. By combining data such as nuclear magnetic resonance hydrogen spectroscopy and elemental analysis, it is proven that the synthesized compound is the designed target product.

### 2.2. Krafft Point

The characteristic of Kraft is the critical dissolution temperature, which is the lowest temperature at which ionic surfactants form aggregates [12,13]. The K_t_ values of CnEO-5 (*n* = 8, 12, and 16) are shown in Table 1.

From the data in the table, it can be seen that the K_t_ values of the three surfactants are all below 0 °C, indicating that they have good solubility. In addition, the K_t_ value gradually increases with the growth of hydrophobic carbon chains, because the longer the length of hydrophobic carbon chains, the lower their solubility in water [14].

### 2.3. Surface Activity

**Figure 3:**
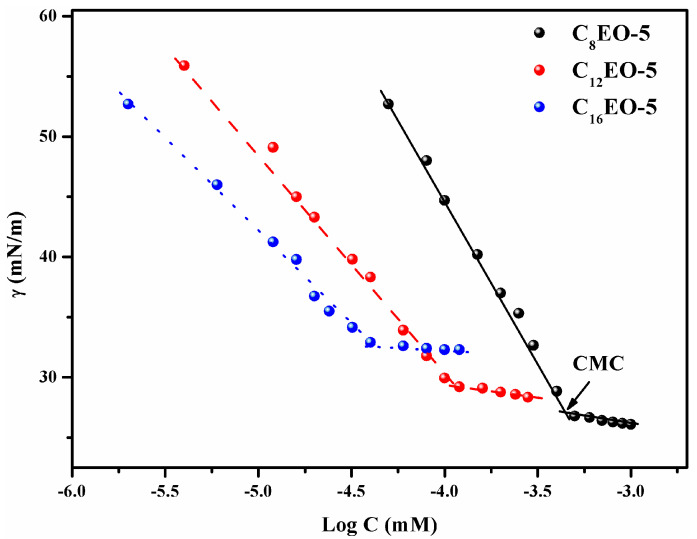
Relationship curve between surface tension and CnEO-5 (*n* = 8, 12, and 16) concentration at 25 °C.

Figure 3 shows the performance of CnEO-5 (*n* = 8, 12, and 16) γ The relationship curve of Log C.

As shown in Figure 3, the surface tension of water rapidly decreases with the increase of CnEO-5 concentration. After reaching the inflection point, its surface tension no longer changes significantly. At this point, CnEO-5 molecules may form aggregates through self-assembly in water, and the concentration corresponding to the inflection point is the critical micelle concentration (CMC) [15]. From Table 2, it can be seen that the CMC values of C8EO-5, C12EO-5, and C16EO-5 are 0.51 mM, 0.107 mM, and 0.032 mM, respectively, which are lower than the CMC values of traditional single chain surfactants (SDS) and other types of anionic non-ionic surfactants. Introducing ethoxyl groups (EO) into ionic surfactant molecules can effectively reduce their CMC [10]. On the one hand, introducing EO groups between hydrophilic and hydrophobic carbon chains can disperse charges, weaken the electrostatic repulsion between surfactant molecules, and lead to a decrease in CMC [10,16].

On the other hand, Wang’s research group [17] and Zhao Guoxi’s research group [18] found that the hydrophilic group EO also has a certain hydrophobicity. The introduction of EO can reduce the free energy of micelle formation, leading to a decrease in CMC. This is basically consistent with the physicochemical properties of the anionic non-ionic surfactants synthesized by Hou’s research group [19]. In addition, the introduction of hydroxyl groups into surfactant molecules can not only insert into the surface of micelles, reduce the electrostatic repulsion between ionic groups, but also form hydrogen bonds with each other, thereby facilitating their aggregation in aqueous solutions [20,21,22].

Furthermore, as shown in Table 2, as the hydrophobic alkyl chain increases, its CMC value gradually decreases. This is mainly due to the stronger hydrophobic interactions between longer alkyl chains [13].

**Table 2:** Surface performance parameters of CnEO-5 and other types of surfactants at 25 °C.

Surfactant	CMC (mM)	γCMC (mN m^−1^)	Гmax/10^−6^ (mol m^−2^)	Amin (nm^2^)	πCMC (mN m^−1^)	pC20
C8EO-5	0.51	26.96	2.35	0.71	45.29	4.29
C12EO-5	0.107	29.69	1.71	0.97	42.51	4.97
C16EO-5	0.037	32.84	1.40	1.19	39.36	5.31
GAES-0805	1.35 a	28.94 a	2.24 a	0.74 a	-	-
SDS	1.63 b	39.0 b	-	-	-	-

a Reference [10]; b Reference [23].

In order to further investigate the adsorption of CnEO-5 molecules at gas-liquid interfaces, this section calculated the maximum saturated adsorption capacity of CnEO-5 molecules based on formulas (1) and (2) (Гmax)Max and the minimum molecular occupied area (*A_min_*) [24,25].

(1) $$\Gamma_{max}=-\frac{1}{2.303nRT}{\left(\frac{\partial_{\gamma }}{\partial_{logC}}\right)}_{T}$$

(2) $$A_{min}=\frac{10^{16}}{N_{A}\Gamma_{max}}$$

Among them, N_A_ is the Avogadro constant; C is the concentration of CnEO-5; partial γ/∂ LogC is the slope of the linear part; γ It is surface tension; 

The values of T and R are 298.15 K and 8.314 J mol^−1^ K^−1^, respectively; *n* = 2 [19].

As shown in Table 2, with the increase of hydrophobic alkyl chain length, the performance of CnEO-5 Г The maximum value gradually decreases, while the Amin value gradually increases. This is mainly because the longer the hydrophobic tail of Gemini surfactants, the easier it is to curl, resulting in a decrease in the stacking density of adjacent molecular side chains, leading to Amin and γ The increase of CMC [15,26,27].

π CMC is generally used to indicate the ability of surfactant molecules to reduce water surface tension. If the π CMC value is higher, it indicates a higher efficiency in reducing water surface tension [28]. The value of π CMC can be calculated using formula (3) [29]:

(3) $$\pi_{CMC}=\gamma_{0}-\gamma_{CMC}$$

Among them, γ CMC is the surface tension at which surfactant molecules begin to aggregate in water; γ 0 is the surface tension of water.

From Table 2, it can be seen that the π CMC value of CnEO-5 decreases with the increase of hydrophobic alkyl chains. Among them, C8EO-5 has the lowest π CMC value, indicating its highest efficiency in reducing water surface tension.

PC20 represents the adsorption efficiency of surfactant molecules at the gas-liquid interface, which can be obtained by Formula (4) [24,30]:

(4) $$pC_{20}=-logC_{20}$$

Among them, C20 represents the concentration of surfactant when reducing the surface tension of water by 20 mN m^−1^.

Generally speaking, the higher the pC20 value, the more likely the surfactant molecules are to adsorb and have a higher efficiency in reducing water surface tension [31]. The experimental results show that C16EO-5 has the highest adsorption efficiency.

### 2.4. Thermodynamic Function of Micellization

**Figure 4:**
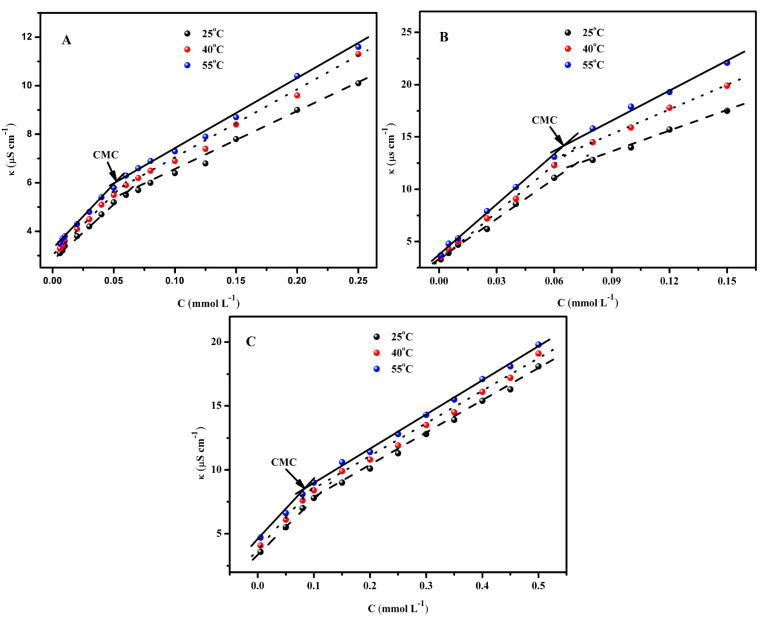
The k vs. C relationship curve at different temperatures, where (**A**) C8EO-5; (**B**) C12EO-5; (**C**) C16EO-5.

By measuring the conductivity curves at different temperatures, the thermodynamic parameters of micellization of CnEO-5 aqueous solutions can be determined. Figure 4 shows the relationship curve between conductivity (k) and concentration (C), where the inflection point of the curve is the CMC of CnEO-5.

The relevant thermodynamic parameters are shown in Table 3. From the data in the table, it can be seen that as the temperature increases from 25 °C to 55 °C, the CMC value in CnEO-5 gradually increases. Generally speaking, the effect of temperature on CMC is mainly reflected in the following two aspects: on the one hand, the increase in temperature weakens the hydration of hydrophilic groups (such as SO^3−^), which is conducive to the formation of micelles; On the other hand, high temperatures can disrupt the structural water molecules near the decomposed hydrophobic alkyl chains, which is not conducive to the formation of micelles [4,20,32]. According to the data in the table, it can be seen that within the experimental temperature range, the latter dominates, so the CMC of CnEO-5 increases with the increase of temperature.

Antiion binding degree (β) It is also an important thermodynamic parameter that reflects the ability of counter ions to bind to micelles, which can be obtained from formula (5) [33]:

(5) $$\beta =1-\frac{S_{micellar}}{S_{premicellar}}$$

Among them, Spremicellar and Smicellar are the slopes before and after the inflection point in the conductivity curve, respectively.

From the data in the table, it can be seen that as the length of hydrophobic alkyl chains increases, β The value gradually increases. This may be related to the charge density of the polar head group [34]. The elongation of alkyl chains not only increases the hydrophobicity of surfactants, but also increases surface charge density, while counterions can bind more tightly with aggregates [15,35]. Additionally, temperature has an impact on β The value also has a certain impact, β The value decreases as the temperature increases. This is mainly because the high temperature weakens the electrostatic interaction between counterions and the surface of micelles [4].

Thermodynamic parameters during micellization process, including standard Gibbs free energy change(Δ*G*°*_mic_*), standard Gibbs enthalpy change(Δ*H*°*_mic_*); Standard Gibbs entropy change(Δ*S*°*_mic_*), etc., can be obtained by formulas (6)–(8) [33,36]:

(6) $$\Delta G_{mic}^{0}=RT(1/2+\beta )\ln X_{CMC}$$

(7) $$\Delta H_{mic}^{0}=-RT^{2}(1/2+\beta )\frac{\alpha_{\ln\chi_{CMC}}}{\alpha_{T}}$$

(8) $$\Delta S_{mic}^{0}=\frac{\Delta H_{mic}^{0}-\Delta G_{mic}^{0}}{T}$$

Among them, XCMC is the molar ratio of surfactant in the solution when CMC is used, XCMC = CMC/55.4 [20].

As shown in the table, all The *ΔG°_mic_* values are all less than 0, indicating that the micellization process of CnEO-5 molecules in water is a spontaneous process [37]. For the same surfactant. The |*ΔG°_mic_*| value increases with the increase of temperature, mainly due to the decrease in hydration around hydrophilic groups caused by the increase in temperature of the surfactant water system, which increases the hydrophobicity of the system. This is also accompanied by an increase in system energy. At this time, the surfactant molecules tend to form micelles, resulting in a decrease in system energy [38,39]. In addition, at the same temperature, the longer the hydrophobic alkyl chain, The negative *ΔG°_mic_* value indicates that the driving force for micellization comes from hydrophobic interactions between alkyl chains [13,15]. The *ΔH°_mic_* values are all less than 0, indicating that the process of CnEO-5 molecules forming micelles in water is exothermic [4,29]. In addition, as the temperature increases, The *ΔH°_mic_* value did not show a significant increase or decrease, indicating that the environment around the hydrophobic chains of CnEO-5 molecules did not change significantly with increasing temperature [40]. The *ΔS°_mic_* values are all positive, indicating that the disorder of the entire system increases during the formation of micelles by surfactant molecules. This is mainly related to the rearrangement of hydrogen bonds formed by CnEO-5 molecules with water and the formation of new “iceberg structures” around the alkyl chains [41]. When they begin to form aggregates, the mutual aggregation between alkyl chains leads to the destruction of the iceberg structure, and the overall chaos of the system increases as a result [42,43]. *TΔS°_mic_* > |*ΔH°_mic_*|indicates that the micellization process of CnEO-5 is mainly entropy driven [40].

### 2.5. Micropolarity

**Figure 5:**
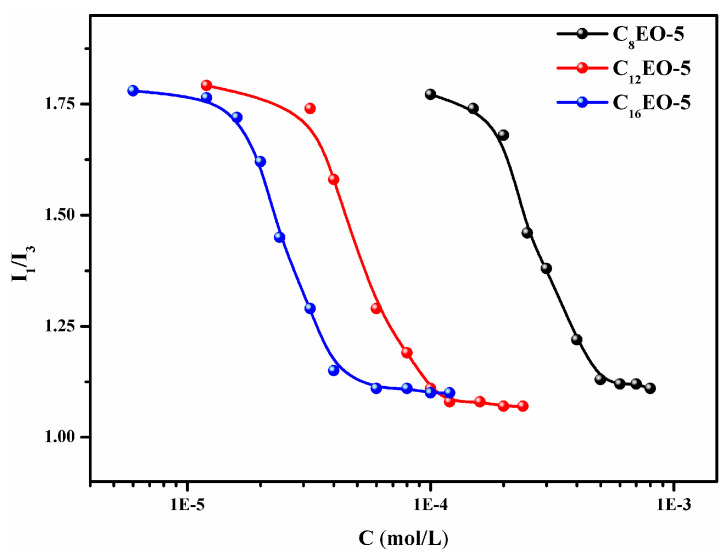
Pyrene intensity ratio (I1/I3) variation curve with CnEO-5 concentration (C).

Generally speaking, the ratio of fluorescence intensity (I1/I3) is highly sensitive to the polar environment in which pyrene molecules (py) are located [4,44]. Therefore, the self-assembly behavior of CnEO-5 molecules in aqueous solutions can be studied by steady-state fluorescence probe method. Figure 5 shows the curve of I1/I3 values versus CnEO-5 concentration.

From the graph, it can be seen that when the concentration of CnEO-5 is low, the I1/I3 value is relatively high, indicating that the py molecule exists in a hydrophilic environment (water) at this time, that is, there are no aggregates such as micelles in the solution; As the concentration of CnEO-5 increases, the I1/I3 value sharply decreases, indicating that the py molecule begins to enter a more hydrophobic environment under the action of surfactants. Finally, due to the dissolution of hydrophobic py molecules in the hydrophobic core of micelles, the I1/I3 values do not show significant changes. This section obtains the CMC value of CnEO-5 through Boltzmann [45] fitting. C8EO-5, C12EO-5, and C16EO-5 are 0.465, 0.112, and 0.042 mM, respectively, which is consistent with the CMC values obtained by the surface tension and conductivity methods mentioned earlier.

### 2.6. Research on Self-Assembly Behavior of CnEO-5

Surfactants with different molecular structures can form different aggregates in aqueous solutions, such as micelles, vesicles, and layers [46]. In 1976, Israelachvili et al. first proposed the concept of critical packing parameter (P) and predicted the morphology of surfactant molecular aggregates based on the magnitude of *p* value [47]. The *p*-value can be obtained by the following formula [21,48]:

(9) $$P=\frac{V_{hydrophobic}}{a_{0}\times l_{0}}$$

(10) $$V=(27.4+26.9\;m)\times 10^{-3}{nm}^{3}$$

(11) $$l_{0}=\left(0.15+0.1265\;m\right)nm$$

Among them, *V* is the volume of hydrophobic carbon chains; Vhydrophobic is the total hydrophobic volume of the entire molecule; L is the chain length of the hydrophobic part of the amphiphilic molecule, and a0 is the area of the hydrophilic group at the head. According to the theory proposed by Israelachvili et al., when *p* ≤ 1/3, the surfactant molecules resemble a cone, with smaller hydrophobic groups and larger hydrophilic groups, resulting in spherical micelles as aggregates; When 1/3< *p* ≤ 1/2, the volume of hydrophobic chain segments increases, making it easy to form other types of micelles, such as ellipsoidal and rod-shaped micelles; When 1/2< *p* ≤ 1, it is easy to form vesicle structures; When *p* > 1, it is easy to form layered and crystalline structures [49].

The calculation results of the critical packing parameters for CnEO-5 are shown in Table 4.

The data in the table indicates that the *p*-value of C_8_EO-5 is greater than 0.5, indicating a tendency to form vesicles in aqueous solutions, while C_12_EO-5 and C_16_EO-5 with longer hydrophobic alkyl chains tend to form micelles.

**Figure 6:**
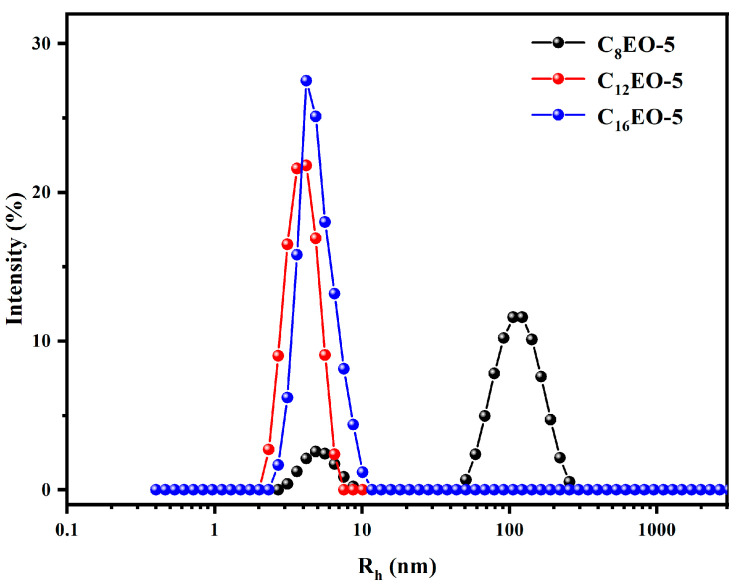
Apparent particle size distribution of CnEO-5 aggregates at 10 CMC.

This section further investigated the aggregation behavior of these three surfactants through DLS, and the results are shown in Figure 6.

As shown in the above figure, there is a clear peak of C_8_EO-5 at 50 nm–100 nm, indicating that C_8_EO-5 molecules may self assemble in water to form larger aggregates (possibly vesicles); However, C_12_EO-5 and C_16_EO-5 exhibit clear peaks in the range of 2 nm–10 nm, indicating that their aqueous solutions may contain a large number of small micelles. The DLS results are basically consistent with the predicted critical packing parameters.

**Figure 7:**
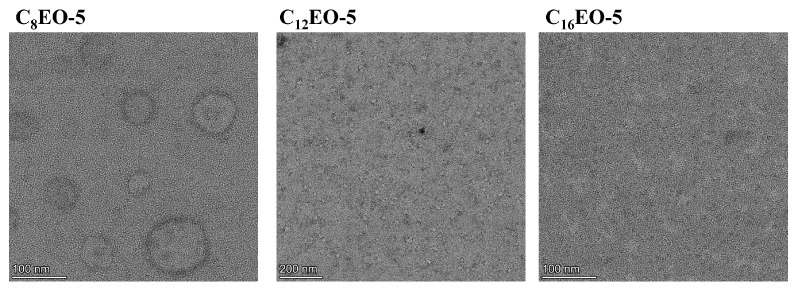
TEM image of CnEO-5 at 10 CMC.

In order to further verify the prediction results of DLS and critical stacking parameters, this section studied the aggregation morphology of CnEO-5 molecules at 10 CMC using transmission electron microscopy, as shown in Figure 7.

**Figure 8:**
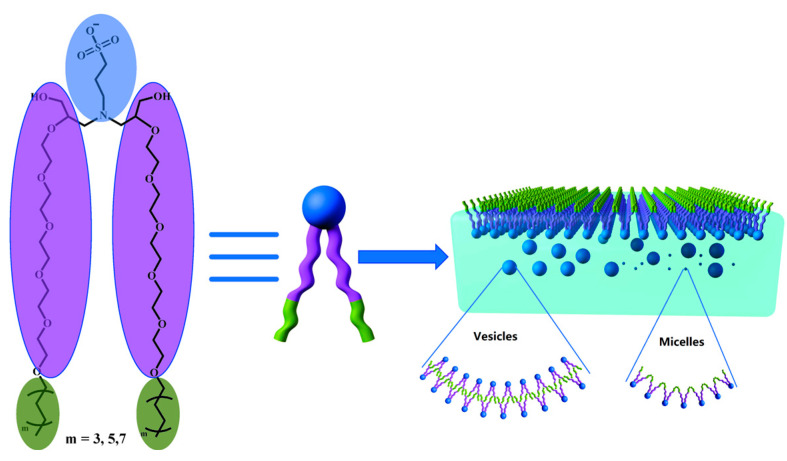
Possible aggregation and adsorption models of CnEO-5 molecules in aqueous solutions.

From Figure 7, it is evident that at 10 CMC, C8EO-5 molecules mainly form larger vesicles in aqueous solution, while C12EO-5 and C16EO-5 form micelles, which is consistent with the results of DLS and *p*-value calculations. In addition, C8EO-5 molecules mainly form vesicles, so their *p*-value range should be between 0.5 and 1. Therefore, the hydrophobic chain l0 of C8EO-5 can be calculated as {(Vhydrophobic/(P × *a*_0_)}, which is 1.02 nm~2.05 nm. This value is significantly smaller than the hydrophobic chain length (3.439 nm) obtained by Formula (11), which suggests that the hydrophobic tail chains of C8EO-5 molecules form vesicles in a tail to tail and staggered manner (as shown in Figure 8).

### 2.7. Foam Performance

**Figure 9:**
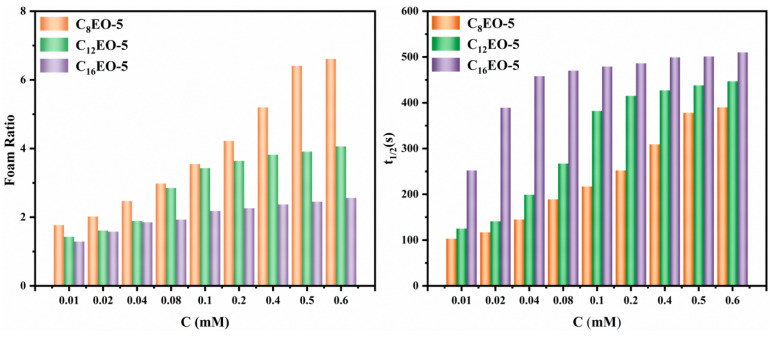
Foam Performance of CnEO-5 (*n* = 8, 12, 16).

Foam ratio (R) and foam half-life (t1/2) are two important indicators to measure the performance of surfactant foam. Generally speaking, the larger the R value, the better the foaming performance [50]; The longer the half-life, the better the stability of foam [51]. The foam performance of CnEO-5 (*n* = 8, 12, 16) is shown in the following Figure 9.

As shown in the figure, the foaming concentration of CnEO-5 gradually increases with increasing concentration, and when it reaches the critical micelle concentration, it no longer significantly increases. In addition, as the number of carbon atoms on the alkyl chain increases from 8 to 16, the R value gradually decreases and the foaming ability gradually decreases. This is mainly because the foaming property of surfactant is related to its surface tension. The lower the surface tension, the less work required to generate foam with the same total surface area, and the better the foaming property [51,52,53]. From the graph, it can also be seen that the half-life time of CnEO-5 increases with the increase of alkyl chain length. The foam rupture is mainly because the liquid film separating the gas becomes thinner from the thick, so the stability of the foam is related to the strength of the formed gas-liquid film. In general, the stronger the hydrophobic effect of surfactant molecules, the more tightly they are adsorbed on the liquid membrane, and the better the stability of foam is [44,54].

## 3. Materials and Methods

### 3.1. Experimental Materials

TBA > 99% Shanghai Aladdin Reagent Co., Ltd. (Shanghai, China). 3-aminopropane sulfonic acid > 98% Shanghai Aladdin Reagent Co., Ltd. (Shanghai, China). Fatty alcohol polyoxyethylene ether 99% Hai’an County petrochemical plant (Hai’an, China). N-hexane > 99% Shanghai Aladdin Reagent Co., Ltd. (Shanghai, China). Ethyl acetate 99% Shanghai Aladdin Reagent Co., Ltd. (Shanghai, China). Sodium hydroxide 99% Shanghai Aladdin Reagent Co., Ltd. (Shanghai, China). Anhydrous ethanol > 99.9% Shanghai Titan Technology Co., Ltd. (Shanghai, China). Acetone > 99.5% Chemical Reagent Co., Ltd. (Shanghai, China). Ether > 99.5% National Pharmaceutical Group Chemical Reagent Co., Ltd. (Shanghai, China). Pyrene > 95% Shanghai Aladdin Reagent Co., Ltd. (Shanghai, China). Copper Mesh, enhanced carbon support film, Shanghai Titan Technology Co., Ltd. (Shanghai, China).

### 3.2. Experimental Apparatus

Nuclear magnetic resonance spectrometer Avance III 500 MHz (Bruckbeisberg GmbH, Switzerland). Automatic Surface Tension Meter Bzy-1 Shanghai balance instrument factory (Shanghai, China). Desktop precision conductivity meter DDJS-308A Shanghai Huyue Ming Scientific Instrument Co., Ltd. (Shanghai, China). Rotary evaporator RV8V Eika (Guangzhou, China) Equipment Co., Ltd. Fluorescent photometer RF-5300PC Shimadzu Enterprise Management (Shanghai, China) Co., Ltd. Particle size analyzer Zetasizer Nano-ZS90 Malvern Instruments, Ltd. (Shanghai, China). High-resolution transmission electron microscope JEM-2100F (JEOL Company, Tokyo, Japan).

### 3.3. Experimental Method for the Synthesis of Anionic Non-Ionic Gemini Surfactant

Fatty alcohol ethoxylates (0.2 mol), tetrabutylammonium bromide (TBAB, 0.016 mol), KOH (0.4 mol) and n-hexane (400 mL) were placed in a 1000 mL three-mouth flask. After stirring at 25 °C for 30 min, slowly add ECH (0.4 mol) dropwise while stirring. After the ECH dropwise addition (about 60 min), the temperature was raised to 45 °C and the reaction was carried out for 12 h. After the reaction, the light yellow filtrate was filtered and the reaction solvent was removed by a rotary evaporator to obtain a crude product. Finally, the compound (II.-1) was recrystallized by the mixed solution of acetone/ethanol with a yield of 76.83%.

**Figure 10:**
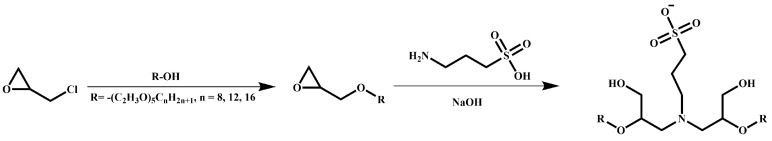
Synthetic route of PKO 15−3(OH)−n (*n* = 12, 14, 16).

3-aminopropanesulfonic acid (0.04 mol), epichlorohydrin (0.085 mol), ultrapure water (110 mL) and ethanol solution (30 mL) were placed in a 500 mL three-mouth flask, stirred for 10 min, and the pH value of the reaction system was adjusted to 9 by 30% sodium hydroxide aqueous solution. The temperature was then raised to 60 °C and the reaction was carried out for 12 h. After the reaction, it is cooled to room temperature and filtered to obtain a filtrate. An appropriate amount of absolute ethanol was added to the filtrate to remove the unreacted 3-aminopropane sulfonic acid, and the crude product (pale yellow liquid) was obtained by filtering and removing the reaction solvent by a rotary evaporator. Finally, anionic Gemini surfactant (CnEO-5, *n* = 8, 12, 16) was obtained by recrystallization of acetone/ethyl acetate mixture solution with a yield of 80.37%. Figure 10 shows the synthetic route of CnEO-5 (*n* = 8, 12, 16).

### 3.4. Experimental Methods for Testing and Characterization

#### 3.4.1. Krafft Point Test

Krafft Point (K_t_) is an important property of ionic surfactants. The solubility of this type of surfactant does not change significantly with the increase of temperature in the lower temperature range, but when the temperature rises to a certain value, its solubility increases rapidly with the increase of temperature, and the temperature at this time is called the Clough characteristic [55]. The specific test method is as follows: first, the surfactant is prepared into a 1 wt% aqueous solution, and then placed in the refrigerator (<0 °C) for 24 h to ensure that the clear and transparent aqueous solution becomes turbid. Finally, the aqueous surfactant solution was removed and placed in a thermostat and heated at a heating rate of 0.1 °C/min until the aqueous surfactant solution changed from turbidity to clear and transparent again, the temperature at which time was recorded, and the above experiment was repeated three times [12,44,56].

#### 3.4.2. Surface Tension Test

The surface tension of the three surfactants was determined by the platinum plate method. Firstly, CnEO-5 aqueous solutions with different concentration gradients were prepared and allowed to stand for 24 h at room temperature, and then the surface tension of different concentrations of aqueous solutions was measured at room temperature, and the relationship curves between surface tension (γ) and logarithm concentration (Log C) were drawn. Each experimental sample was measured in triplicate and averaged. In addition, the surface tension of ultrapure water was measured at room temperature at 72.20 mN/m.

#### 3.4.3. Conductivity Test

The conductivity of different concentrations of CnEO-5 aqueous solution was determined by DDJS-308A conductivity meter at 25 °C, 45 °C and 65 °C, respectively, and the conductivity meter was calibrated according to the measurement of the conductivity of the known concentration of potassium chloride solution. Before the test, the electrode is washed three times with ultrapure water to remove impurities from the electrode surface, and after cleaning, it is immersed in a standard solution.

#### 3.4.4. Steady-State Fluorescence Test

The steady-state fluorescent probe method is a method used to determine the critical micelle concentration of surfactants, in which pyrene (py) probes are commonly used hydrophobic probes [57]. The fluorescence spectra of different concentrations of CnEO-5 aqueous solutions were determined by Shimadzu RF-5300PC fluorescence spectrophotometer, where the concentration of py was 10^−6^ mol/L [4]. The specific test conditions are as follows: the excitation wavelength is 335 nm, the emission spectrum scanning range is 350 nm~450 nm, the emission slit width is 2 nm, and the excitation slit width is 5 nm [20].

#### 3.4.5. Dynamic Light Scattering Test

The hydrodynamic diameter of CnEO-5 molecules forming aggregates in aqueous solution (10 CMC) was determined by a nanoparticle size potentiometer (Zetasizer Nano-ZS90). Among them, the scattering angle is 90° and the experimental temperature is 25 °C. The aqueous solution of the surfactant was filtered with a 0.45 mm filter head to remove some of the undissolved material [58].

#### 3.4.6. Transmission Electron Microscope

Determine the aggregation behavior of CnEO-5 molecules in aqueous solution at 10 CMC using negative staining method [38]. The specific testing steps are as follows: first, prepare a certain concentration of CnEO-5 aqueous solution (10 CMC) and let it stand at room temperature for 24 h. Then, take a certain amount of CnEO-5 aqueous solution and drop it onto the copper mesh used for TEM testing. After 5 min, use clean filter paper to remove the liquid from the copper mesh and stain it with a 2 wt% aqueous solution of phosphotungstic acid. Similarly, after 5 min, use clean filter paper to remove excess phosphotungstic acid aqueous solution from the copper mesh and dry it naturally at room temperature for 12 h.

#### 3.4.7. Foam Performance Test

The foam performance of the surfactant is measured by the oscillation method. The half-life (t1/2) of the foam and the foam ratio (R) are used to measure the stability and foaming ability of the foam [24]. The R value can be obtained by the ratio of the initial foam height after oscillation to the initial solution height. T1/2 is the time required to separate half of the liquid from the initial foam through self drainage. The specific testing method is as follows: First, prepare different concentrations of CnEO-5 aqueous solutions (50 mL) and let them stand at room temperature for 24 h. Then, take out 5 mL into a stoppered measuring cylinder and vigorously shake for 20 s [59].

## 4. Conclusions

A series of novel anionic non-ionic Gemini surfactants were synthesized by introducing EO into hydrophobic alkyl chains using fatty alcohol polyoxyethylene ether with different hydrophobic chain lengths, 3-aminopropane sulfonic acid, and epichlorohydrin as raw materials through substitution and ring opening reactions. The molecular structures of these three surfactants were determined through 1H NMR and elemental analysis. The surface tension test results show that CnEO-5 has high surface activity and is higher than traditional single chain surfactants and structurally similar anionic non-ionic Gemini surfactants. Additionally, thermodynamic parameters (e.g., *ΔG°_mic_ ΔH°_mic_ ΔS°_mic_* et al. indicate that CnEO-5 molecules are exothermic and spontaneous during the micellization process. DLS, *p*-values, and TEM results indicate that anionic non-ionic Gemini surfactants with shorter hydrophobic chains (such as C_8_EO-5) tend to form larger vesicles in aqueous solutions, which are formed in a tail to tail and staggered manner; Negative non-ionic Gemini surfactants with longer hydrophobic chains (such as C_12_EO-5, C_16_EO-5) tend to form small micelles.

## Figures and Tables

**Table 1:** K_t_ values of CnEO-5 (*n* = 8, 12, and 16).

Surfactant	K_t_ (°C)
C_8_EO-5	<−5 °C
C_12_EO-5	<−2 °C
C_16_EO-5	<0 °C

**Table 3:** Thermodynamic parameters during the micellization process of CnEO-5 molecules.

Surfactant	*T* (°C)	CMC (mmol L^−1^)	β	Δ*G°_mic_* (kJ·mol^−1^)	Δ*H°_mic_* (kJ·mol^−1^)	ΔS*°*_mic_ (kJ·mol^−1^·K^−1^)	TΔS*°*_mic_ (kJ·mol^−1^)
C_8_EO-5	25	0.525	0.473	−37.33	−7.41	0.1004	29.93
	40	0.645	0.451	−37.81	−7.99	0.09523	29.82
	55	0.715	0.420	−38.08	−8.48	0.09020	29.60
C_12_EO-5	25	0.108	0.487	−32.17	−6.73	0.08532	25.43
	40	0.124	0.459	−32.49	−7.21	0.08073	25.28
	55	0.142	0.435	−32.83	−7.72	0.07652	25.11
C_16_EO-5	25	0.0278	0.501	−35.99	−13.76	0.07456	22.230
	40	0.0410	0.483	−36.13	−14.91	0.06776	21.22
	55	0.0486	0.458	−36.45	−15.95	0.06247	20.50

**Table 4:** Critical Stacking Parameters P and Other Physical and Chemical Parameters.

Surfactant	V_hydrophobic_ (nm^3^)	*l*_0_ (nm)	*p*
C_8_EO-5	0.7268	3.439	0.60
C_12_EO-5	0.8344	3.945	0.44
C_16_EO-5	0.942	3.945	0.40

## Data Availability

Data is contained within the article.
